# Trigone ventricular glioblastoma multiforme with trapped temporal horn: A case report

**DOI:** 10.3389/fonc.2022.995189

**Published:** 2022-09-13

**Authors:** Lei Liu, Shaozhen Wang, Xuetao Dong, Yaodong Liu, Liudong Wei, Linghong Kong, Qingjun Zhang, Kun Zhang

**Affiliations:** ^1^ Department of Neurosurgery, Chui Yang Liu Hospital Affiliated to Tsinghua University, Beijing, China; ^2^ Department of Pathology, Chui Yang Liu Hospital Affiliated to Tsinghua University, Beijing, China

**Keywords:** glioblastoma multiforme, intraventricular, trigone tumor, trapped temporal horn, case report

## Abstract

**Background:**

Intraventricular glioblastoma multiforme (GBM) is extremely rare, especially in the trigone region. This report presents a case of trigone ventricular GBM with trapped temporal horn (TTH).

**Case presentation:**

A 59-year-old woman was admitted to our department with a 1-month history of rapidly progressive headache, nausea, and weakness in the right lower extremity. Head non-contrast computed tomography and enhanced magnetic resonance imaging (MRI) revealed a trigone ventricular mass lesion with TTH and heterogeneous enhancement. The lesion was found 18 months ago as a small asymptomatic tumor mimicking ependymoma. This neoplasm was removed subtotally through the right parieto-occipital approach guided by neuroendoscopy. A ventriculoperitoneal shunt was subsequently performed to relieve TTH. The final pathological diagnosis was GBM. Unfortunately, 36 days after the first surgery, the patient died due to her family’s decision to refuse therapy.

**Conclusion:**

This rare case shows that GBM should be considered in the differential diagnosis of trigonal tumors. In this case, the tumor possibly originated from the neural stem cells in the subventricular zone. Patients with intraventricular GBM have a worse prognosis, and careful follow-up and early surgery for small intraventricular tumors are necessary, even for those with ependymoma-like radiological findings.

## Introduction

Glioblastoma multiforme (GBM) is a common primary intracranial malignancy that accounts for approximately 25% of all brain tumors in adults (1, 2) but rarely develops within the ventricles, especially the trigone region. Despite current advanced therapeutic strategies combining surgery, radiation, and chemotherapy, patients with GBM survive for a median time of approximately 15 months (3, 4). The tumor site plays an important role in the prognosis of patients with GBM, and lesions that are closer to the lateral ventricles lead to shorter survival times and higher rates of distant relapse (5–9). Generally, intraventricular GBMs are asymptomatic at an early stage and are identified when they develop as advanced lesions, leading to space-occupying effects or obstructive hydrocephalus. Therefore, it was difficult to distinguish their origins. This report presents an extremely rare case of trigone GBM with trapped temporal horn (TTH) first diagnosed as a small asymptomatic tumor in the body of the right lateral ventricle mimicking ependymoma. This case helps explain the pathogenesis of intraventricular glioma.

## Case presentation

**Figure 1:**
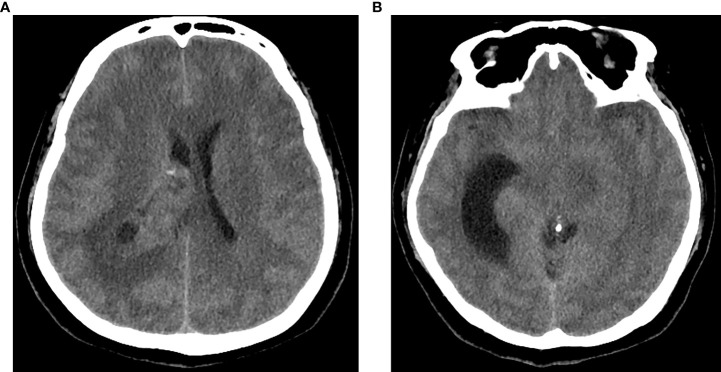
CT of the head showed a right intraventricular solid and cystic mass lesion **(A)** with trapped temporal horn and periventricular edema **(B)**.

**Figure 2:**
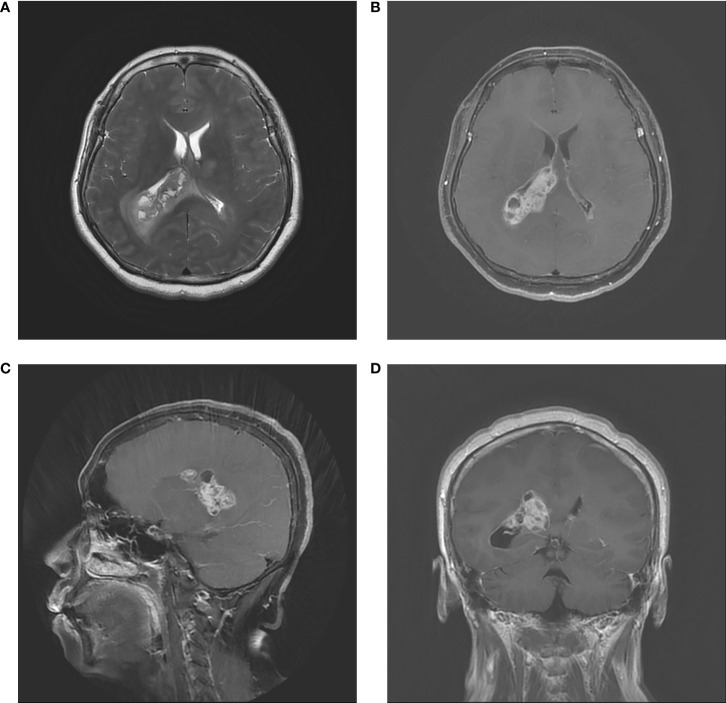
Preoperative MRI. **(A)** Axial T2–weighted image. **(B)** Axial contrast MRI. **(C)** Coronal contrast MRI. **(D)** Sagittal contrast MRI. The tumor had a diameter of 50 mm in and located in the trigone region of the right lateral ventricle. MRI showed infiltrative, irregular borders, inhomogeneous contrast enhancement, and rim enhancement.

A 59-year-old woman was admitted to our department with 1-month history of rapidly progressive headache, nausea, and weakness in the right lower extremity. Physical examination revealed slight hemiparesis in the right lower limb (muscle power grade IV). A non-contrast computed tomography (CT) revealed a right lateral ventricular mass lesion and TTH, as well as diffuse brain edema (**Figure 1**). Subsequent magnetic resonance imaging (MRI) verified the lesion site in the trigone with temporal horn expansion. The space-occupying lesion was solid, cystic, brim enhancing, and heterogeneous. The maximum diameter of the lesion was 5 cm (**Figure 2**).

**Figure 3:**
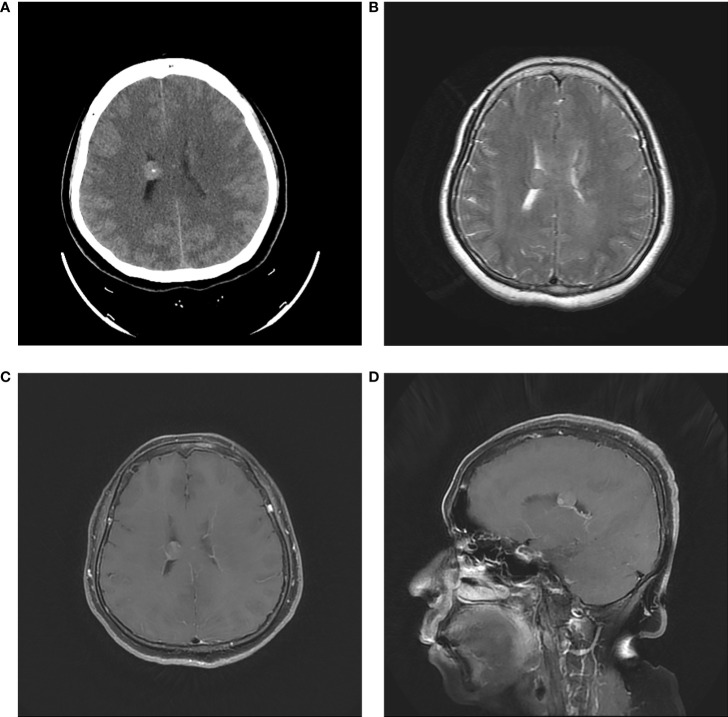
Initial head CT revealed a 1-cm round high-density lesion and punctate calcification in the center **(A)**. T2-weighted MRI showed the regular tumor located in the body of right lateral ventricle **(B)**. Axial and sagittal contrast MRI showed minimal enhancement **(C, D)**.

In fact, the tumor had been detected incidentally by CT and MRI when the patient visited our hospital 18 months ago. CT revealed a 1-cm round high-density lesion in the body of the right lateral ventricle and punctate calcification in the center. Minimal enhancement was observed on enhanced MRI (**Figure 3**). Based on imaging characteristics, ependymoma was the most probable diagnosis. The patient chose observation treatment. At the sixth month of follow-up in another hospital, no change in tumor size was noted, and she had not been followed since then until she developed progressive headache.

**Figure 4:**
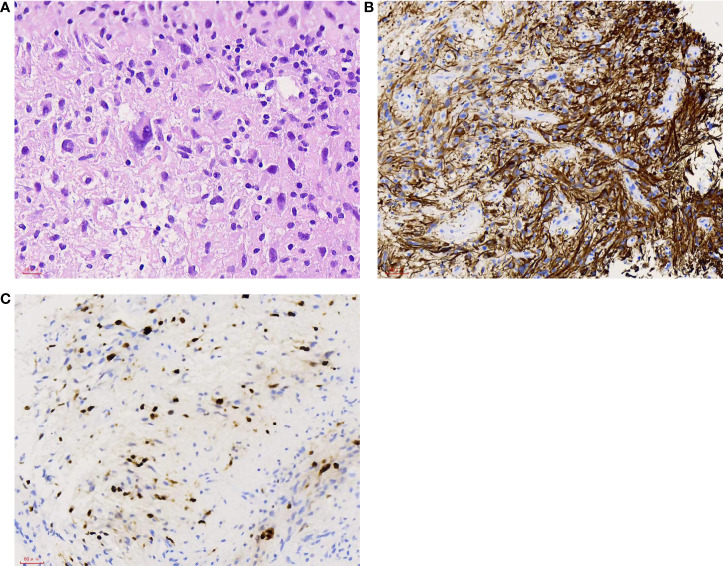
**(A)** Tumor cells with atypical nuclei. H&E, 400×. **(B)** Most tumor cells show positive staining. GFAP, 200×. **(C)** High proliferation index with approximately 20% of the tumor cells showing nuclear staining. Ki67 200×.

A right parieto-occipital surgical approach was used, and surgery was guided by neuroendoscopy. The tumor appeared grayish and predominantly soft and was so infiltrative that no obvious interface between the ependymal layer and lesion was differentiated. Due to the large tumor volume and deep location, subtotal resection was achieved. Histopathological examination of the tumor revealed endothelial proliferation, atypical nuclei, and necrosis. Immunohistochemistry demonstrated strong positivity for glial fibrillary acidic protein (GFAP), negativity for IDH1, and Ki67 index of 20% (**Figure 4**). The final pathological diagnosis was consistent with glioblastoma IDH wild type (2021 WHO CNS5). The patient recovered unevenly, the TTH was more distinct than that preoperatively, and external ventricular drainage through the temporal horn and ventriculoperitoneal shunt were performed one after another. The patient remained comatose postoperatively. After the patient’s family was informed about the disease prognosis, they chose to pursue palliative care and comfort measures. However, the patient died 36 days after the first surgery.

## Discussion

GBM is a common primary intracranial malignancy that accounts for approximately 25% of all brain tumors (1, 2). Although it can be found anywhere in the central nervous system, intraventricular GBM is relatively rare, and trigone GBM is extremely rare. To the best of our knowledge, there have been no more than six clear cases of GBM in the trigone (10–13).

Tumors that arise from the ventricular wall or structures within the ventricle are regarded as primary ventricular tumors, such as choroid plexus papilloma, choroid plexus carcinoma, ependymoma, and meningioma. Neoplasms that originate from the surrounding brain parenchyma and grow into the ventricle are regarded as secondary ventricular tumor. Astrocytoma, subependymal giant cell astrocytoma, GBM, and mixed glial neuronal tumors consist the group (14, 15). In our case, the initial pathological diagnosis was ependymoma because of its regular shape and calcification. However, one and a half years of history has met the natural history of glioblastomas, since glioblastomas usually show rapid progression. We believe that the final histopathological diagnosis of GBM is the result of a transition from lower grade glioma.

Lee et al. illustrated that neuroglial cells of the septum pellucidum or fornix may be the origin of intraventricular GBM. Particularly, the fornix and limbic systems are closely connected to the ventricular system (1). Kim et al. emphasized that the subependymal zone and pluripotent stem cells within it are important (16). Strong evidence in the past 10 years has indicated that glioma stem cells may originate from neural stem cells (NSCs) existing in the adult subventricular zone (SVZ) (17, 18). Lee et al. suggested that NSCs in the SVZ were probably the cell of origin, which contains the driver mutations of GBM and provided molecular genetic confirmation of this issue (19). Referring to this case, we also believe that the lesion originated from NSCs in the SVZ. It initially originated from the SVZ at the body of the lateral ventricle and then gradually infiltrated the trigone region.

Radiologically, ependymomas, central neurocytomas, choroid plexus tumors, and some metastases can be found in calcifications (16). Hence, ependymoma is the most likely diagnosis. Unfortunately a spectroscopy MRI was not performed to further exclude the rare possible diagnosis of a high-grade gliomas at the time of the first diagnosis. It seems that not all intraventricular GBMs present typical imaging features of high-grade gliomas, such as infiltrative, inhomogeneous enhancement, and irregular brim (1, 10, 20). Park et al. reported a case of well-circumscribed, minimally enhancing GBM of the trigone (10). Patnaik et al. reported a case of intraventricular GBM mimicking meningioma (21).

The majority of lateral ventricular neoplasms usually grows slowly and does not cause symptoms until they are large enough to induce compression of adjacent eloquent structures or obstructive hydrocephalus (10). In our case, the headache attributed to local hydrocephalus was in accordance with a large intraventricular tumor. The most common tumors in the trigone in adults are meningiomas, ependymomas, metastatic tumors, and astrocytomas (11, 21). Tumors rarely involve only the temporal horn and meningiomas are the most common (12). We first reported a rare case of trigone GBM with TTH.

Patients with GBM have a dismal prognosis, and they always survive for no more than 2 years. Relapse *in situ* or distant recurrence is nearly inevitable, despite the utilization of standard therapeutic strategies, including gross total resection and concurrent chemoradiotherapy (22, 23). As far as GBM location is concerned, tumors located near the lateral ventricle are more aggressive than those located far from the lateral ventricle. Increased tumor volume, increased distant relapse, and decreased survival independent of resection extent indicate increased malignancy (5, 7, 8, 24). Fyllingen et al. considered that the distance from the tumor enhancement margin to the center of the third ventricle could be a practical prognostic factor in patients with GBM. Corpus callosum, basal ganglia, and other central tumor locations were correlated with an overall survival of < 6 months (25). Among the reported cases of trigone GBM, Park et al. also adopted parietooccipital surgical approach to resect a well-circumscribed, minimally enhancing GBM of the trigone without hydrocephalus and a near-total removal was accomplished. Whole-brain radiation and chemotherapy were subsequently performed; the patient remained neurologically intact at 2-year follow-up (10). Other authors all adopted transcortical approach to remove trigone GBM but not mentioned the prognosis. Owing to the deep tumor location and ill-defined border, subtotal resection was achieved; however, the TTH and periventricular edema worsened postoperatively. The patient showed short-term improvement after a ventriculoperitoneal shunt (through the temporal horn) but died.

## Conclusions

Intraventricular GBM is relatively rare. This report presents a rare case of trigone ventricular GBM with TTH. Therefore, GBM should be considered in the differential diagnosis of trigone tumors. This may arise from NSCs in the SVZ. Moreover, the prognosis of patients with intraventricular GBM is worse, indicating that intensive follow-up and early surgery for intraventricular tumors are necessary, even for those that show ependymoma-like radiological findings.

## Data availability statement

The raw data supporting the conclusions of this article will be made available by the authors, without undue reservation.

## Ethics statement

Written informed consent was obtained from each individual for publication of potentially identifiable images or data included in this article.

## Author contributions

Conception and design: QZ and KZ. Data acquisition: KZ and LK. Analysis and interpretation of data: KZ and LK. Article drafting: LL and KZ. Reviewed submitted version of the manuscript: all authors. Critical revision of the article: all authors. Approved the final version of the manuscript on behalf of all authors: KZ. Study supervision: QZ and KZ. All authors contributed to the article and approved the submitted version.

## Acknowledgments

The authors thank the members of their research group for useful discussions and hard work. We would like to thank Editage (www.editage.com) for English language editing.

## Conflict of interest

The authors declare that the research was conducted in the absence of any commercial or financial relationships that could be construed as a potential conflict of interest.

## Publisher’s note

All claims expressed in this article are solely those of the authors and do not necessarily represent those of their affiliated organizations, or those of the publisher, the editors and the reviewers. Any product that may be evaluated in this article, or claim that may be made by its manufacturer, is not guaranteed or endorsed by the publisher.
